# Postsynaptic Receptors for Amyloid-β Oligomers as Mediators of Neuronal Damage in Alzheimer’s Disease

**DOI:** 10.3389/fphys.2012.00464

**Published:** 2012-12-20

**Authors:** Margarita C. Dinamarca, Juvenal A. Ríos, Nibaldo C. Inestrosa

**Affiliations:** ^1^Centro de Envejecimiento y Regeneración, Departamento de Biología Celular y Molecular, Facultad de Ciencias Biológicas, Pontificia Universidad Católica de ChileSantiago, Chile

**Keywords:** Aβ oligomers, postsynaptic receptors, neuroligin-1, synaptotoxicity, Alzheimer’s disease

## Abstract

The neurotoxic effect of amyloid-β peptide (Aβ) over the central synapses has been described and is reflected in the decrease of some postsynaptic excitatory proteins, the alteration in the number and morphology of the dendritic spines, and a decrease in long-term potentiation. Many studies has been carried out to identify the putative Aβ receptors in neurons, and is still no clear why the Aβ oligomers only affect the excitatory synapses. Aβ oligomers bind to neurite and preferentially to the postsynaptic region, where the postsynaptic protein-95 (PSD-95) is present in the glutamatergic synapse, and interacts directly with the *N*-methyl-D-aspartate receptor (NMDAR) and neuroligin (NL). NL is a postsynaptic protein which binds to the presynaptic protein, neurexin to form a heterophilic adhesion complex, the disruption of this interaction affects the integrity of the synaptic contact. Structurally, NL has an extracellular domain homolog to acetylcholinesterase, the first synaptic protein that was found to interact with Aβ. In the present review we will document the interaction between Aβ and the extracellular domain of NL-1 at the excitatory synapse, as well as the interaction with other postsynaptic components, including the glutamatergic receptors (NMDA and mGluR5), the prion protein, the neurotrophin receptor, and the α7-nicotinic acetylcholine receptor. We conclude that several Aβ oligomers receptors exist at the excitatory synapse, which could be the responsible for the neurotoxic effect described for the Aβ oligomers. The characterization of the interaction between Aβ receptors and Aβ oligomers could help to understand the source of the neurologic damage observed in the brain of the Alzheimer’s disease patients.

## Introduction

Alzheimer’s disease (AD) is the most common form of dementia with a high prevalence rate among the aging population, and recent estimates suggest that it affects about 35 million individuals worldwide (Querfurth and LaFerla, 2010). Clinical manifestations are characterized by loss of selective cognitive functions, particularly memory, loss that begins early in the disease progression (Selkoe, 2002; Ballard et al., 2011). These traits are accompanied by neuropathological features observed in postmortem AD brains. Studies of AD brains have revealed the presence of the classical neuropathological hallmarks, neurofibrillary tangles, and senile plaques (Haass and Selkoe, 2007). These senile plaques are aggregates of amyloid-β peptide (Aβ) which are deposits in brain areas involved in cognitive functions and it is assumed that they initiate a pathological cascade that results in synaptic dysfunction, synaptic loss, and neuronal death (Haass and Selkoe, 2007; Cerpa et al., 2008). Aβ spontaneously self aggregates into multiple coexisting physical forms. One of them consists of oligomers (ranging from dimers to dodecamers) which coalesce into intermediate assemblies. Fibrils, which arrange themselves into β-pleated sheets to form the insoluble fibers of advanced amyloid plaques. Soluble oligomers and intermediate amyloid are the most neurotoxic forms (Klein et al., 2001; Kayed et al., 2003). It is known that synapses are lost during Alzheimer’s and there is a high correlation between synaptic loss and cognitive decline observed in AD patients (Serrano-Pozo et al., 2011). Aβ oligomers selectively block long-term potentiation (LTP) and acutely disrupt cognitive function after infusion into the central nervous system (CNS; Haass and Selkoe, 2007; Cerpa et al., 2008). They also bind with a punctuate pattern to postsynaptic excitatory pyramidal neurons but not to GABAergic neurons (Lacor et al., 2004) and lead to synaptic loss (Wei et al., 2010). In both AD patients and animal models of the disease, synapse loss is greatest near to senile plaques, indicating a link between amyloid pathology and synaptotoxicity *in vivo* (Lacor et al., 2004; Wei et al., 2010). There is considerable evidence that specificity of Aβ binding at neuronal surfaces and synapses requires membrane proteins, and several candidate receptors that may have an important role in expression of Aβ neurotoxicity have been postulated as potential targets for Aβ (Table 1). The objective of this article is to review the interactions between soluble Aβ oligomers and specific neuronal postsynaptic receptors in the context of Aβ-mediated synaptic disruption and neurotoxicity.

**Table 1:** **Different putative Aβ oligomer receptors in neurons**.

Synaptic protein	Type of Aβ aggregate	*K*_d_	Reference
α7AChR	42 Monomer	4.1 pM	Wang et al. (2000)
α4β2AChR	42 Monomer	30 nM	
AChE	12–48 fragment	–	Alvarez et al. (1997)
Fz5-CRD	40 Monomer	0.105 μM	Magdesian et al. (2008)
Fz5-CRD	42 Monomer	–	
Fz5-CRD	ADDL	–	
Fz5-CRD	Fibril	–	
PrPc	Oligomer	92 nM	Lauren et al. (2009)
NMDAR	ADDL	–	De Felice et al. (2007)
TNF-R	40 Monomer	4.2 pM	Li et al. (2011)
α2β1 integrin	40 Monomer	–	Wright et al. (2007)
αVβ1 integrin	40 Monomer	–	
APP	Fibril	–	Lorenzo et al. (2000)
NL-1	Monomer/oligomer	0.75 μM	Dinamarca et al. (2011)

*The Table presents the main putative receptors that bind or interact with different types of Aβ aggregates, in some cases the dissociation constant (*K*_d_) is included. In other cases, it shows protein that are not receptors, for example AChE and the APP, both proteins interacts with Aβ aggregates*.

## Glutamate Receptor as a Mediator of Aβ Toxicity

Synaptic degeneration, including loss of synapses in the brain and the consequent impairment in synaptic plasticity, is the best morphological correlate of cognitive impairment in clinical AD (Terry et al., 1991; Sze et al., 1997). It has been demonstrated that cognitive impairments in learning and memory are related to degenerative synaptic changes produced by the presence of soluble Aβ oligomers in vulnerable brain regions such as the hippocampus (Lambert et al., 1998; Walsh and Selkoe, 2004; Almeida et al., 2005; Haass and Selkoe, 2007). Glutamatergic neurotransmission has an essential role in synaptic transmission and neuronal plasticity processes associated with learning and memory (Bard and Groc, 2011). Impairment in glutamatergic neurotransmission signaling has been described as an important factor in AD pathology (Snyder et al., 2005; Yamin, 2009; Proctor et al., 2011). Glutamate receptors consist of two classes: ionotropic and metabotropic, members of both classes have been implicated as putative neuronal receptors that mediate Aβ oligomer neurotoxicity.

## *N*-Methyl-D-Aspartate Receptors

*N*-methyl-D-aspartate receptors (NMDARs) located at the postsynaptic density of dendritic spines play key roles in glutamatergic transmission and synaptic plasticity in the CNS. These receptors are important for induction of LTP and synaptic transmission (Snyder et al., 2005; Bordji et al., 2011; Proctor et al., 2011). A number of studies have shown that Aβ can affect the function of NMDARs and abolish induction of NMDAR-dependent LTP at the neuronal plasma membrane (Snyder et al., 2005; Shankar et al., 2007). Aβ binds to NR1 and NR2B subunits of the hippocampal neurons NMDAR (Lacor et al., 2004, 2007). Aβ oligomers induce the endocytosis of NMDA receptors by a α7-nicotinic acetylcholine receptor (α7-nAChR)-dependent manner (Snyder et al., 2005). Also α7-nAChRs are involved in deregulation of NMDA signaling pathways (Roselli et al., 2005; Shankar et al., 2007). In synaptosomes, Aβ oligomers co-immunoprecipitated with NMDAR subunits and partially (50%) co-localize, in hippocampal slices (De Felice et al., 2007; Deshpande et al., 2009; Renner et al., 2010; Rönicke et al., 2011). However, it is unclear the domain to which Aβ directly binds to NMDAR subunits. The NR2B subunit of NMDAR has been implicated in regulating the action of Aβ oligomers by increased intracellular calcium into dendritic spines resulting in reduced dendritic spine and synaptic density that led to early synaptic dysfunction (Shankar et al., 2007). Also, the stimulation of NR2B by Aβ oligomer triggers the activation of MAPK and the subsequent down-regulation of CREB (Li et al., 2011). Interestingly, relatively low doses of NMDA-antagonists have been reported to reverse Aβ-induced synaptic disruption (Li et al., 2011; Rönicke et al., 2011). In addition, Aβ has been shown to decreased formation clusters at the postsynaptic membrane, reducing levels of scaffolding proteins such as PSD-95, which has an important role in synaptic plasticity and stabilization of glutamate receptors located at excitatory synapses. Also, this dysfunction has been associated to an enhancement of NR2A activity (Liu et al., 2010). Moreover, in hippocampal neurons Aβ oligomers disrupts axonal transport initiated by NMDAR-dependent mechanisms and mediated by the enzyme glycogen synthase kinase-3β (GSK-3β; Decker et al., 2010). Therefore, different pathways related to NMDARs, are in direct or indirect way involved in the neurotoxicity mediated by the Aβ oligomers.

## Metabotropic Glutamate Receptor 5 (mGluR5)

The GluR5 subtype of metabotropic glutamate receptors located at the neck of dendritic spines has important regulatory roles in excitatory synaptic transmission at hippocampal synapses, including modulation of LTP and potentiation of NMDAR-mediated calcium influx (Renner et al., 2010). A recent study identified mGluR5 as a novel neuronal receptor target for Aβ using single particle tracking of quantum dot labeled Aβ oligomers on hippocampal neurons, and examining their interactions with mGluR5 receptors. Membrane-bound Aβ oligomers accumulated at synaptic space, where they progressively aggregated to form large non-mobile clusters through lateral diffusion. Pathological clusters of Aβ formed complexes with mGluR5 receptors, leading to decreased mobility of mGluR5 and causing their aberrant accumulation at the postsynaptic membrane. This was followed by calcium deregulation, synaptic disruption, and loss of NMDARs, suggesting that their role in early synaptic impairment is induced by Aβ (Renner et al., 2010). Interestingly, mouse hippocampal neuronal cultures from mGluR5-knockout mice revealed an approximately 80% decrease in Aβ binding to the neuronal surface and a loss of NMDARs from the cell surface (Renner et al., 2010). mGluR signaling is down-regulated and desensitized in the frontal cortex of AD patients and has been shown to correlate with AD related neuropathological changes. Moreover, mGluR5 shares a functional relationship with NMDAR, in particular because it relates to development of NMDA-dependent LTP and learning (Rammes et al., 2011). Furthermore, chronic activation of mGluR5 increases NMDA-dependent Aβ neurotoxicity, whereas its inhibition shows neuroprotective effects against Aβ excitotoxic processes (Rammes et al., 2011). Thus, there is persuasive experimental evidence that Aβ oligomers are able to influence glutamatergic transmission by affecting both NMDAR and mGluR5 receptors. Therefore antagonists of these receptors have become promising targets for the treatment of AD. Memantine (a non-selective NMDAR antagonist) is currently being used in clinical practice for the treatment of patients with moderate to advanced AD (De Felice et al., 2007).

## p75 Neurotrophin Receptor in Aβ Oligomer Neurotoxicity

p75 is a transmembrane glycoprotein and low-affinity nerve growth factor (NGF)-receptor, member of the tumor necrosis factor receptor superfamily, which binds all neurotrophins with similar affinity but different kinetics (Dechant and Barde, 2002), neurotrophins have diverse functions in the CNS, initially synthesized as precursors (proneurotrophins), they are cleaved to produce mature proteins, which promote neuronal survival and enhance synaptic plasticity by activating Trk receptor tyrosine kinases. The broad spectrum of biological activities exerted by the neurotrophins results from their ability to bind and activate two structurally unrelated receptor types, the p75-NTR, and the three members (in mammals) of the Trk receptor family of tyrosine kinases (Dechant and Barde, 2002). The various functions of p75-NTR depend on the type of ligand bound to it, the cell type in which it is expressed and the presence or absence of Trk receptor. p75 induces the neurotrophin NGF mediated survival in neuronal cells expressing TrkA. Moreover, neurons expressing p75-NTR without co-expressing Trk underwent apoptosis upon NGF treatment (Dechant and Barde, 2002). In addition, p75-NTR mediates a range of neurobiological functions, including cell fate, axon guidance, and modulation of neurite outgrowth (Barker, 2004). It has been demonstrated that all neurotrophins that activated p75, without co-activation of the relevant Trk co-receptor, mediated apoptosis in hippocampal neurons. Thus, proneurotrophins and Aβ can induce p75-mediated apoptosis in hippocampal neurons since they do not bind or activate Trk receptors. The p75-mediated Aβ cytotoxicity mechanism involves the downstream activation of p75 intracellular death domain (Diarra et al., 2009). The death domain in turn activates G0 subtypes of G protein, which leads to c-Jun N-terminal kinases (JNK) phosphorylation. Although there are many pathways hypothesized, most of the sources found the terminal outcome of this process to be cell death (Coulson, 2006). These findings were confirmed by another group who found that hippocampal neurons became apoptotic after Aβ_1-42_ treatment (Sotthibundhu et al., 2008). Therefore, hippocampal neurons undergo neurotrophin-dependent p75-mediated apoptosis in the absence of Trk co-activation. Ligands that activate p75 but not Trk, such as Aβ and proneurotrophins, might contribute to the pathogenesis of AD. However, it remains to be determined which of these ligands has the most impact in disease pathogenesis. It is also worth determining whether reducing level of such ligand can alleviate pathogenesis of AD.

## α7-nAChR as a Mediator of Aβ Toxicity

Since the 1970s it has been proposed that the cholinergic system plays an important role in the pathogenesis of AD. Early studies indicate that in patients who have the disease, there is a decrease in the number of cholinergic neurons (Davies and Maloney, 1976; Whitehouse et al., 1982), a decrease in acetylcholine synthesis (Sims et al., 1983), a reduced number of cholinergic receptors in the cortex (White et al., 1977; Kellar et al., 1987), reduced acetylcholinesterase (AChE) activity (Perry et al., 1978a), and increased butyryl-cholinesterase (BuChE) activity (Perry et al., 1978b; Bartus et al., 1982). These discoveries led to propose the “cholinergic hypothesis of AD,” in which the lacks of acetylcholine was responsible for triggering AD pathology, therefore specific drugs were selected by their capacity to increase acetylcholine, including AChE and BuChE inhibitors or nAChR agonists (Bartus et al., 1982; Mangialasche et al., 2010). Interestingly those neurons which express abundant α7-nAChR are more vulnerable to damage by Aβ_1–42_, the most neurotoxic form of Aβ, this could be due to their strong physical interaction (Wang et al., 2000), and/or to the receptor internalization allows with the Aβ peptide bound to it (Nagele et al., 2002). This would explain the accumulation of large amounts of the Aβ peptide in the perikaryon of pyramidal neurons of the forebrain observed previously of Aβ deposition (D’Andrea et al., 2002). Also of interest is the association of the α4 and α7 subunits of the nicotinic receptor to the senile plaques present in AD brains (Wevers et al., 1999). Moreover, it has been suggested that the α7-nAChR expressed in the smooth muscle of brain blood vessels in AD patients, allows a greater deposition of Aβ_1–42_ peptide responsible for the amyloid angiopathy present in AD brains (Clifford et al., 2008). In *Caenorhabditis elegans*, it has been shown that the Aβ peptide expressed in the nematode muscle triggers a mislocalization and a synaptic dysfunction of the ACR16, the homolog receptor protein of α7-nAChR in the worm (Rebolledo et al., 2011).

Currently there is evidence in the literature supporting the idea that the Aβ peptide acts as an agonist or antagonist of nicotinic receptors (Buckingham et al., 2009). In fact, Aβ_1–42_ interferes the signaling of different nicotinic receptors in *Xenopus* oocytes (Lamb et al., 2005). In rat hippocampal slices, acetylcholine currents mediated by nicotinic receptors of interneurons are inhibited when they are incubated with nM concentrations of the Aβ peptide (Pettit et al., 2001). Supporting its role as agonist, Dougherty et al. (2003) shows a calcium increase after exposure hippocampal presynaptic nerve endings to the Aβ peptide. Early studies indicated also in the α7-nAChR expressed in *Xenopus* oocyte that small amounts of Aβ peptide (10–100 pM), would act as an agonist, however larger amounts (100 nM) produces a desensitization of the receptor (Dineley et al., 2002). On the other hand, it has been observed that the Aβ peptide can affect downstream nAChR receptor signaling. One example is when Aβ peptide through α7-nAChR triggers a down-regulation of the ERK2/MAPK pathway, which has been associated with memory formation (Dineley et al., 2001). Supporting this data, post mortem analysis of AD and Down syndrome brains showed an increase in the phosphorylation of ERK protein (Swatton et al., 2004). In addition other studies suggest that *tau* phosphorylation, triggered by the Aβ peptide is mediated through the α7-nAChR by the activation of GSK-3β (Hu et al., 2008; Bitner et al., 2009) and also to the activation of the JNK and ERK (Wang et al., 2003; Schliebs and Arendt, 2011). The Aβ peptide also produces an increase in the phosphorylation of Akt (p-Akt) in acute treatment of primary neurons in culture, in a manner NMDAR and α7-nAChR dependent. However, chronic exposure to the Aβ peptide leads to a decrease of p-Akt levels. In old transgenic AD model (APPswe/PSEN1ΔE9), the active enzyme levels were considerably lower than in wild-type mice (Abbott et al., 2008). Akt phosphorylation has been associated with a pro-survival function, and alterations in this pathway are associated with an increased in the severity of AD (Lee et al., 2009).

## Prion as an Amyloid Receptor

Cellular prion protein is a glycosyl phosphatidylinositol (GPI)-anchored cell surface protein (Varela-Nallar et al., 2006). Recently, it has been suggested that the binding of Aβ oligomer to cellular prion protein (PrPc) is essential for synaptic toxicity reflected in the loss of LTP (Lauren et al., 2009). Moreover, ablation of PrPc enhances cognitive function in transgenic mice overexpressing mutant amyloid precursor protein (APP) gene (APPswe and PS1ΔE9) preventing premature death of neurons and memory impairment (Gimbel et al., 2010). However, other reports questioned these findings because the absence of PrPc did not prevent Aβ oligomer-mediated synaptic toxicity or cognitive impairment (Balducci et al., 2010; Calella et al., 2010; Kessels et al., 2010). However, these results confirm the physical interaction between Aβ and PrPc, however it remains unclear the role of PrPc in this pathology, for this, more studies are necessary to establish whether PrPc is essential for the Aβ oligomer neurotoxicity. Recently, it has been reported that neuronal cell death induced by synthetic Aβ oligomer was prevented by reducing or eliminating PrPc, or blocking the binding between PrPc and Aβ oligomer using either a PrPc antibody or a decoy PrPc peptide (Kudo et al., 2011). Additionally, it has been described that PrPc participates in Aβ transcytosis across the blood-brain barrier (Pflanzner et al., 2012). In summary, the interaction between PrPc and Aβ is very interesting, because both are pathogenic proteins responsible of neurodegenerative diseases and this particular relationship reinforce the idea of crosstalk between protein misfolded in neurodegeneration process (Morales et al., 2010; Forloni and Balducci, 2011).

## Neuroligin-1 as a Target for Aβ Oligomers

Postsynaptic neuroligins, and their related presynaptic receptors neurexins (NRXs), are two families of synapse-specific adhesion molecules critically involved in establishing CNS connectivity (Dean and Dresbach, 2006; Südhof, 2008). NL is a transmembrane protein type I with three domains, an extracellular cholinesterase-like domain, a single transmembrane helix, and a cytoplasmic C-terminal domain that contains a type I PDZ-binding motif which interacts with the third PDZ domain of PSD-95 protein (Scholl and Scheiffele, 2003). However, not just the known NLs PDZ domain is important in NLs function, recently a new non-PDZ domain on the cytoplasmic tail has been described as an essential element for the postsynaptic functional effects of NLs at excitatory synapses (Shipman et al., 2011). There are four types of NLs: NL-1 is enriched in glutamatergic excitatory synapses, while NL-2 is present in GABAergic inhibitory synapses. On the other hand, NL-3 and NL-4 have been found in both synapses; also, NL-4 has been found in extraneuronal tissues (Scheiffele et al., 2000; Dean and Dresbach, 2006). Initially NLs were identified as ligands for β-neurexin; thereby a role in mediate cell–cell adhesion was proposed. Disruption of NLs–NRX signaling precipitates a broad range of cognitive deficits, including motor, learning, and social impairments (Blundell et al., 2010; Dahlhaus and El-Husseini, 2010; Dahlhaus et al., 2010), and specific NL and NRX mutations have been implicated in autism (Südhof, 2008). NLs were shown to be sufficient to induce the formation of new functional presynaptic terminals *in vitro*, suggesting a key role in the specification and consolidation of the synapse (Dean and Dresbach, 2006). Considerable evidence links NL–NRX signaling to synapse initiation. In an assay of artificial synaptogenesis, NL-1 expressed in non-neuronal cells is sufficient to induce presynaptic differentiation in contacting axons of co-cultured neurons (Scheiffele et al., 2000). Moreover, NL-1 overexpression in primary neuronal cultures potently increases synapse densities and can increase spontaneous miniature postsynaptic current frequencies, while NL-1 knockdown inhibits normal synaptogenesis (Dean and Dresbach, 2006; Shipman et al., 2011). NL-1 deletion strongly impairs neurotransmission while minimally impacting synapse densities, suggesting that NL-1 may primarily contribute to synapse function and not formation (Blundell et al., 2010). On the other hand, it is known that synapses are lost during Alzheimer and there is a high correlation between synaptic lost and cognitive decline observed in AD patients (Terry et al., 1991; Walsh and Selkoe, 2004; Serrano-Pozo et al., 2011). Growing evidence from numerous laboratories suggest that soluble Aβ oligomers, more than monomers or fibrils, are responsible for Aβ cytotoxicity (Haass and Selkoe, 2007; Li et al., 2011). As we have been discussed along this review, several receptor proteins have been proposed to be capable of binding various forms of Aβ, thereby inducing its cellular effecs. We had presented both biochemical and physicochemical evidence *in vitro* indicating that NL-1 binds to Aβ oligomers, this interaction occurs in the extracellular domain of NL-1, suggesting that NL-1 could be a putative target for Aβ oligomers at excitatory synapse. No effect was observed with NL-2 which is a specific NL form for inhibitory synapses. Given the important roles of NL-1 as an adhesion protein on the postsynaptic membrane, the interaction with Aβ oligomers can be relevant for the stability and the maintenance of synaptic transmission (Dinamarca et al., 2011). Moreover, the fact that NL-1, a postsynaptic protein forms an heterophilic adhesion complex with Neurexin, a presynaptic protein, indicates that any disruption of this interaction might affects the integrity of the excitatory synaptic contact.

## Conclusion

**Figure 1:**
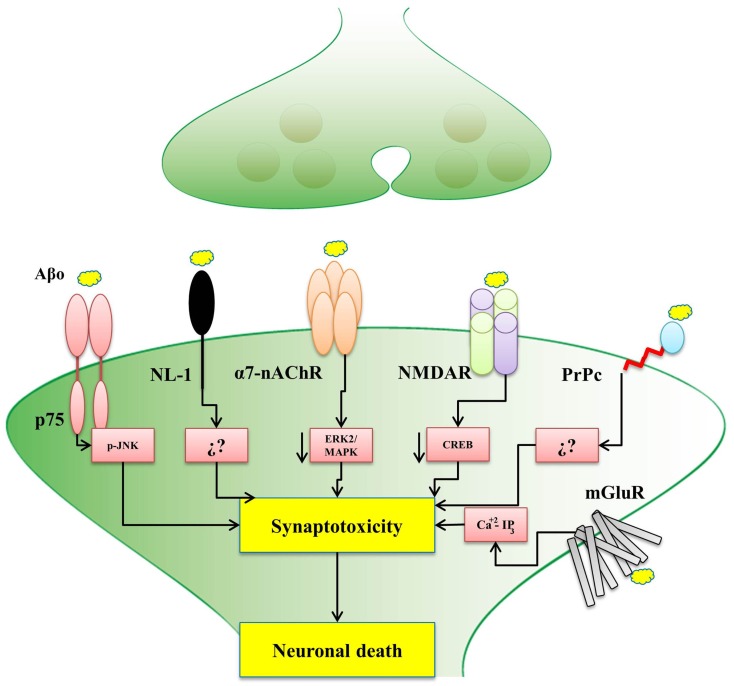
**Postsynaptic receptors for Aβ oligomers affect different signaling pathways**. The scheme shows that the binding of Aβ oligomers to different postsynaptic receptors might trigger synaptotoxicity and consequent neuronal death by activating different signaling pathways, as indicated, only in the case of NL-1 (neuroligin-1) and the PrPc (cellular prion protein), the precise pathway is not known. p75 (p75 receptor of neurotrophin), α7-nAChR (α7-nicotinic acetylcholine receptor), NMDAR (*N*-Methyl-D-aspartate receptor), and mGluR (metabotropic glutamate receptors), the signaling pathway is already known. In the case of the α7-nAChR, recent studies from our laboratory also implicated the *Wnt* signaling pathway.

The evidence presented in this review, for the interaction among Aβ oligomers with different synaptic proteins and receptors is a key contribution to the understanding of the development of early AD (Figure 1). In fact, a depth understanding of how these Aβ-interacting synaptic proteins influence synaptic plasticity and memory processes would help to elucidate the mechanisms by which soluble Aβ oligomers cause neuronal receptor dysfunction and triggers the disease. Hopefully, all these studies will set an scenario where new therapeutic strategies emerge to help to develop new an effective treatments for AD.

## Conflict of Interest Statement

The authors declare that the research was conducted in the absence of any commercial or financial relationships that could be construed as a potential conflict of interest.
